# Modern Treatment of Eclampsia, Considered from a Medical Standpoint*Read before Section on Obstetrics and Gynecology, New York Academy of Medicine, May 29, 1896.

**Published:** 1896-08

**Authors:** S. Marx

**Affiliations:** Instructor in Obstetrics, New York Post-graduate Medical School; Assistant Surgeon New York Maternity


### MODERN TREATMENT OF ECLAMPSIA, CONSIDERED FROM A MEDICAL STANDPOINT.*

By S. MARX, M. D.,

Instructor in Obstetrics, New York Post-graduate Medical School; Assistant Surgeon New York Maternity.

The pathogenesis of eclampsia is surrounded by a mist of doubt. The multiplicity of theories advanced as to its cause stamps it at once as a disease about which we know little or nothing. The uncertainty as to the primary factors inducing eclampsia naturally very materially modifies our course as to prognosis and treatment.

That albuminuria bears a certain relation to eclampsia no one gainsays, for it has been our experience that, in nearly 95 per cent. of the cases of threatened or already present convulsions, albumin is present in the urine. The condition is certainly a toxemia, whether of the auto-infective type, as from an accumulation in the blood of a toxic material, be this urine, feces, or both, or due to the presence of creatin and creatinin, or again, as has been lately advanced, a leucomaine poison; or on the other hand, of the heterogenetic type having a bacillary origin, with its bacteria toxins; or, finally, depending upon a chemical basis as a result of anomalies of oxidation, if we may so choose to call it, as, for instance, a carbaminic-acid poison. Yet clinical experience teaches that eclampsia is a condition closely allied to and largely associ-

*Read before Section on Obstetrics and Gynecology, New York Academy of Medicine, May 29, 1896.

ated with disturbances of renal secretion, and that the surest method of treatment is to cause a return of diuresis. Experimental evidence with the urine of eclampsia shows it three times less poisonous than that from a healthy woman; and again, that eclampsic blood or its serum shows a hypertoxicity more than three times stronger than normal blood serum. It is safe to say that no one distinct and specific factor comes into play in all cases; for have not all physicians seen women, apparently perfectly well, attacked with eclampsia, which came like a thunder-clap from a clear sky, in whom every measure failed to check the fatal course of the malady, the whole scene lasting from 24 to 36 hours? Certainly this picture—and one not overdrawn—stamps the disease as one of an acute infection of the most malignant type. And, on the other hand, are there not mild cases running their course in a perfectly benign manner, and, in spite of procrastination, getting well with little or no deviation from the normal state? But, at best, eclampsia is a very dangerous condition, and one not to be thought lightly of in any case.

Generally speaking, a woman with an albuminuria must be regarded with suspicion, nay, apprehension; and the physician who does not attempt to attack the trouble in its incipiency does not do his full duty by his patient. Physicians have always been, even to this day, as derelict in examining the urine of pregnancy as they are in measuring pelves. How many women would still be alive to-day, and spared to their families, had an examination of the urine been made regularly monthly or bi-monthly! It is often too late to treat a patient when in eclampsia, for at least 45 per cent. of patients die in this condition. It is not safe to predict that the first attack may not be fatal, or that the next convulsion may not be the last; for it is the absolute uncertainty of the disease which makes the condition all the more dangerous and the prognosis so thoroughly bad. An albuminuria or a threatened uremia can be remedied, or at least the dangerous symptoms held in abeyance; but how gloomy the prognosis, when convulsions have already occurred, or when a fat embolus of the lung or an apoplexy of the brain occurs as a result! Personally, I wish to go on record in stating that eclampsia is as absolutely preventable as is puerperal sepsis. Nay, more so—

since even among the best and the most careful, sepsis will occasionally creep in. Not so with eclampsia; for if men were taught, or, if taught, would remember, that regular examination of urine should be made, and is as essential during the whole of pregnancy as asepsis is during labor, fewer eclampsias would occur, and, *ergo*, fewer women and children be lost. Men depend too much upon the presence or absence of albuminuria in determining the condition of a pregnant woman. Even in the presence of constitutional symptoms, as œdema, headaches, and so forth, the physician rests satisfied so long as no albumin is present in the urine, and declares his patient safe, and yet, while temporizing in a position of ignorant security, he is dealing with one of the most malignant states and fatal cases, just where there is uremia without albuminuria, a condition that can be well denominated "urinemia." These cases are not so very fatal *per se*, but they *are* so treacherous because they are not recognized. It is just here that the attempt will be made to elucidate symptoms as they occur in the toxemia of pregnancy without albuminuria.

If I can only impress my readers with the fact that urea estimation, going to show a diminished excretion of urea, is of far greater importance than the mere presence of albumin, one of the objects in writing this article has been attained. To make this statement concise, it will be emphatically said that in a large majority of cases albumin is simply a danger-signal, and serves one, as the red flag, as a warning of an incipient catastrophe; on the other hand, its absence means absolutely nothing in the presence of certain symptoms. It is my belief that urinemia is due to complete occlusion of one ureter, from compression by the fœtal head, or from extra uterine causes, as for instance, a prior existing pelveo-peritonitis; the bands of adhesions thus formed completely shutting off the flow of urine from one kidney. This causes a retention and a damming back of the urine behind the occlusion into the pelvis of the kidney, and is thus absorbed, the other organ functionating normally. It will thus be readily understood that the absorption is very slow, the system at large acclimating itself, as it were, to the slow but gradual absorption, not only of the watery elements of the urine, but of its solid constituents. The condition occurs as a rule in the primipara in whom there occurs early and complete adaptation of the head about four weeks before labor. The flow of urine is suddenly diminished, and there is frequent urination. The specific gravity is high and albumin is absent. Urea estimation reveals the same very materially diminished. In a few cases the presence of a hydronephrotic tumor has been noted. The presence of œdema is characteristic. It is *erratic*. At times the patient is bloated to a marked degree, and then in a few hours it has entirely disappeared. Again there are temporary local œdemas, which disappear as quickly as they arise. It is this symptom which is as typical as any, and, to my mind, characteristic of the condition. On account of the insidious absorption of urine there is noted a very gradual systemic intoxication. So gradually are the symptoms developed that they escape attention, yet they are sufficient for the careful observer to determine that a toxemia is present. The convulsions before labor are very rare; as a rule the explosion occurs during or immediately after labor. This is followed by an increased discharge of urine. With this comes an increased excretion of urea, and with it albumin appears for the first time, gradually increasing, and finally diminishing in proportion as the urea increases.

When I come to take up the treatment of eclampsia I am at once cognizant of the fact that I am dealing with the most difficult subject in the therapeusis of obstetrics. It is with a good deal of diffidence and hesitancy that the same is approached; but what follows is not based upon theoretical deductions, but is the result of actual clinical experience; in other words, practical therapeutics at the bedside. In the large majority of the cases drugs will be of little assistance in staying the course of the disease, for all that may be done will not and does not control the symptoms. I am in favor of temporizing so long as the symptoms, especially those that refer to the urine, rapidly disappear and the condition improves; but when in any case, after vigorous, conscientious and scientific treatment, the symptoms do not improve, or, at least, remain stationary, there is only one method of treatment by which the attendant may hope to save our patient, and that is emptying the uterus. I am so pessimistic about the toxemia of pregnancy or the pre-eclampsic state that in no case would I be willing to wait longer than three days, in the absence of improvement either local or constitutional, for fear of the awful consequences which might endanger the patient by waiting. In deciding upon the course to take I depend solely upon the amount of urea excreted in 24 hours, and not upon the increase or decrease of the albuminuria or the presence or absence of œdema. To temporize longer means not only to invite convulsive seizures with their enormous mortality rate, but also the almost inevitable death of the fœtus. It is a well-known fact that eclampsia is rare after the uterus is emptied, for it is in less than 5 per cent. of the cases that the first convulsion occurs after labor. Then, why in these cases temporize with drugs, when there is almost everything to be gained and practically nothing lost by interfering by surgical means? I have always regretted when I temporized; and in a few cases where surgical interference was instituted early, and things went wrong, there has always been the feeling that had I been even more active and terminated labor sooner, the result might have been more successful. The secret of success in treating these cases surgically is early interference, rapidly emptying the uterus, under deep but short chloroform narcosis. I shall attempt to treat the subject only from a clinical standpoint, which will help me to more clearly define my position so far as therapeutics, medically speaking, is concerned.

I.—Acute toxemia depending on heart disease.

II.—Acute toxemia depending on an acute exacerbation of a chronic nephritis.

III.—Acute toxemia depending on acute degenerative kidney changes.

IV.—Acute toxemia, in which neither albumin nor casts appear in the urine—a true urinemia.

I. ACUTE TOXEMIA DEPENDING ON HEART-DISEASE.—Properly, the subject under the heading No. I. does not come within the scope of this paper, but the writer has so often seen albumin and casts and symptoms referable to kidney disturbance occur with this condition that he cannot refrain from referring to it. From a prophylactic standpoint, a woman with a confirmed heart-disease, valvular or otherwise, should never marry if she wishes to bear children. The prognosis is almost always bad, should pregnancy occur under these conditions. Should pregnancy occur, absolute rest for the greater part of the day should be insisted on, combined with heart tonics, as digitalis, strophanthus, sparteine, etc. They should be given uninterruptedly, alternating one with the other for a longer or a shorter period, according to the effects produced. So long as compensatory disturbances do not occur—in other words, so long as the heart's rhythm is good and regular and the general condition appears satisfactory—nothing is to be done but to wait and watch. But when once this compensation ceases, when the rhythm becomes or has a tendency to become rapid and irregular, or if the compensation persists, and yet albumin and casts appear with a diminution of either urea or the urinary secretion, do not try tentative measures nor temporize, but empty the uterus at once. I do not hesitate to elect the induction of labor under chloroform narcosis, since it is my experience that the strain of a prolonged labor minus an anesthetic is far too great and much too dangerous to warrant me in withholding a full anesthetic narcosis. The danger is not so great during labor, but after labor it is first that the exhaustion becomes manifest. In six cases I have now recorded, a free administration of nitroglycerin by the needle, beginning before the termination of labor and given with a free hand, from $\frac{1}{50}$ to $\frac{1}{25}$ grn. every hour or two, combined with hot saline enemata, has saved all my patients. It is also allowable to give hypodermatic injections of caffeine or one of its salts, which are all very soluble, in doses of from three to five grn. every hour. Where cerebral symptoms are present, they are contraindicated. Further, it should be remembered that caffeine, having a cumulative effect, symptoms of sharp cerebral irritation may follow, when the exhibition is continued too long. These symptoms are characterized by intense restlessness and pronounced insomnia. Further, strychnine $\frac{1}{30}$ to $\frac{1}{15}$ grn. every hour or two, combined with oxygen inhalation, will do much to tide these patients over the critical period. But they must be exhibited early to anticipate the almost inevitable condition which follows after labor, to wit, collapse. To wait until the danger is already present is to waste time and to sacrifice life, but to anticipate and successfully repel it is our object.

II. TOXEMIA DEPENDING ON AN ACUTE EXACERBATION OF A CHRONIC NEPHRITIS.—This is one of the most important conditions coming under observation. What holds in reference to prophylaxis in heart disease holds doubly good in chronic nephritis, since a patient with renal disease is in greater and more constant danger all the time; for pregnancy will not have advanced very far, according to my own experience, when marked nephritic symptoms of a very acute type will become manifest by a rapid increase of albumin, blood, casts, and a rapid increasing œdema. It is useless and hardly possible to prescribe these patients absolute rest in bed and an exclusive milk diet for any length of time, since they, though chronic sufferers, are still human and will rebel; again, a vigorous and strict *regime* will certainly in time lower their vitality; a condition by all means to be avoided. In looking around some years ago for a method of treatment suitable to this class, it appeared to me that what was wanted was a vaso-motor dilator, as well as a measure which, while increasing the flow of urine, was not a direct kidney irritant. The first indication could be filled by such vaso-motor dilators as chloral, chloroform, KI, small doses of opium, the nitrites, etc.; but these did not materially cause diuresis, and were objectionable, for obvious reasons too numerous to mention. It was not until nitroglycerin was tried that I felt satisfied that the drug had been found to fill every indication. It is ideal cerebral vaso-motor dilator inducing a condition opposite to that found in incipient uremic states, for it is known that an intense cerebral anemia is supposed to be the immediate cause of eclampsic seizures. In small, continuous doses it causes fullness of the head, due to the brain function being exalted by temporary congestion; lowers arterial tension (which in chronic nephritis is of great value, since high tension states are the rule), due to its action upon the muscular coats of the arteries. It increases the flow of urine, possibly on account of its stimulating the heart and dilating the arterioles in the kidneys. From a clinical standpoint I have found repeated small doses, short of producing physiological effects—from $\frac{1}{100}$ to $\frac{1}{150}$ grn. evry two, three, to four hours,—given almost continually throughout pregnancy, of such great value that I should hate to refrain from using it. Nitroglycerin I have used a large number of times. One patient whom I recall to mind, with an old chronic nephritis, suffered severely and was completely water-logged during one pregnancy, in spite of a strict milk diet, etc., and followed by eclampsia; in the next pregnancy, about six weeks after she was out of bed again, she was allowed a mixed diet and continuous doses of nitro-glycerin, taking in all about seven hundred tablets during the pregnancy, and going to term without any anasarca, gave birth to a living child without marked symptoms and without eclampsia. All the patients were allowed plenty of regular out-door exercise, and, what is of great importance, a liberal diet, and meat once a day; insisting on keeping the bowels freely open and on drinking a large amount of water. No other medicines are to be taken. I have met with but one failure, and here the patient, carelessly exposing herself to very in-clement weather, developed symptoms of an intensely acute toxemia, which rapidly passed off upon emptying the uterus. These measures, in my hands, can be honestly recommended as giving the most happy results. I do not claim to have carried all the patients to term, but what is claimed is that I have kept them in a very favorable condition until that time when it was deemed advisable to elect the induction of premature labor, for the reasons that (1) I could depend upon the child living and thriving outside of the uterus, and (2) to avoid the unpleasant symtoms which might possibly arise during the last month of pregnancy. As a rule, though, the vast majority of pregnant nephritics went to term and were delivered in a natural manner, without any untoward symp-toms. When in any case the pulse appeared full and bounding during labor, the patient was allowed to bleed freely after the second stage, *i. e.*, I took no measure to cause contraction of the uterus, but rather favored its relaxation, the so-called uterine venesection, until the pulse became soft and compressi-ble.

III. ACUTE TOXEMIA, DEPENDING UPON ACUTE DEGENERATIVE KIDNEY CHANGES; THE ACUTE NEPHRITIS OF PREGNANCY.—This class of cases includes the most frequent form of toxemia seen by the practitioner. It is as a rule characterized by frank and outspoken symptoms. Its timely treatment will do much to alleviate symtoms, modify the course of the disease, and materially influence the prognosis. The presence of albumin and casts in the urine or of constitutional symptoms should make the physician apprehensive, and cause the patient to go to bed at once, and to stimulate every emunctory to excessive action.

To influence (1) the bowels, it must be seen to that the patient has numerous watery movements every day, not only for the purpose of cleaning out the bowels and to check toxic absorption from that source, and further not only to act on the liver in such a manner as to keep that organ above suspicion as a breeder of toxins, but to deliberately deplete the system and rid it of the poison supposed to be circulating in the blood. I have, as a rule, depended on large doses of calomel and jalap (15 grn. each) and followed it every day by large doses of Rubinat or Hunyadi water, even to the extent of hypercatharsis. Where obstinate constipation is present or prompt action is required, elaterium $\frac{1}{8}$ grn. every hour till catharsis occurs, or croton oil, both of which may simply be placed on the tongue, when their action becomes manifest in a short time. Where for any reason the patient was unable to take medicines by the mouth, it has been my custom to give either high enemata of saturated solution of salines, which act promptly as active hydragogue cathartics, or equally reliable dram doses of a saturated solution of salines hypodermatically. Under other conditions the hypodermic use of salines has answered the purpose admirably on more than one occasion.

To act (2) on the skin is the next consideration. I have found nothing better than either the hot steam bath, given in bed, applied intermittently or continually; or twice a day a very hot full bath, gradually increasing the temperature of the water as hot as can be borne, and allowing the patient to remain for a half-hour or more, the whole tub being covered with heavy woolen blankets, thus giving a sort of vapor bath. The patient is then lifted out, blanket and all, placed in bed, and diuresis is thus further favored. The unpleasant cerebral effects produced may be mitigated by the ice-cap or cold cloths to the head. Besides this, internally there may be used the spirit of Mindererus, or, better still, the sweet spirits of nitre, which not only acts on the skin, but is a mild diuretic, and of great importance as a nitrite, consequently a vaso-motor dilator. As to jaborandi and its alkaloid, pilocarpine, I am absolutely and radically opposed to it from every standpoint. It is admitted that it acts as a certain and powerful diaphoretic, yet it does so at the expense of urinary secretion, lesssening that function very materially. Its heart-depressant effects alone would deter me from countenancing its exhibition, for I have seen such profound collapse from its use as to make me an "over-and-above board" enemy of the drug. Its powerful action on the glands of the mouth and respiratory organs is such as to make the accumulation of mucus in the respiratory tract a decided objection to its use.

To directly influence (3) the urinary secretion there is a large list to choose from—first and foremost, (*a*) *Dietary:* It is essential that these patients take only that class of food which is blandest to the kidney, yet assists in increasing diuresis, and is easy of assimilation, viz.: milk, buttermilk, kumyss, matzoon. My preference is for milk, taken in large amounts, but not hastily swallowed. Where it causes indigestion, it should be taken by the tablespoonful and eaten. I have been accustomed to add to each glass of milk 1 dr. to ½ oz. doses of the sugar of milk, a powerful diuretic and pleasant to take, recommended by the French school and with which there has always been satisfaction. (*b*) *Water:* It is very essential that these patients drink as much water as possible, to stimulate not only the skin, but the kidneys; preference being for a mild saline or pure spring water, viz., Buffalo Lithia or Poland water. Rectal injections of a physiological salt solution, 1 to 2 pints, a number of times a day act exceedingly well, not only as a food, but as a direct urinary stimulant. (*c*) As to *drugs*, their number is legion. Their action should lay in one direction, and, where well chosen, they should not act directly upon the kidney, which is an acutely inflamed organ under these conditions, but indirectly by stimulating the heart and at the same time lowering tension, in order to provoke diuresis.

The refrigerant diuretics of the soda class (acetate, citrate, etc.), with an infusion of buchu or triticum, stand first; abstaining from the use of potash salts, since they are all powerful cardiac depressants. The usual formula given under these conditions, containing some preparation of digitalis, is, to my mind, absolutely contraindicated, since digitalis, possibly on account of its digitoxin, is not only a decided and direct renal irritant, but the drug itself increases vascular tension. Its use is not questioned in chronic conditions, but I object decidedly to its use in acute nephritis. The customary administration of the time-honored tinct. ferri chlor. I object to for the reason that not only the iron, but the amount of alcohol it contains—while it may possibly influence the anemia of the nephritic state, and this is questionable—is too much of a kidney irritant to do anything but harm. Among the drugs of unquestioned value as true heart tonics and diuretics there will be mentioned caffeine 1 grn. to 5 grn., or one of its soluble salts in double the dose every 2 to 3 hours. Nitroglycerin from $\frac{1}{50}$ grn. to $\frac{1}{150}$ grn., sulphate of sparteine ¼ grn. to ½ grn., or the symptomatic remedies acting as vaso-motor dilators—nitrite of soda, chloral, small doses of opium, etc. Of strychnine I am a little wary in cases of high tension pulse, since its action increases arterial tension, but in patients having low tension pulses it is a very valuable remedy.

Of the lately much lauded veratrum viride, while I am free to grant that veratrum is a symptomatic remedy of the greatest value, yet I question its true curative and therapeutic worth in cases demanding its use. It has been used extensively, but in the face of conditions demanding its administration —*i. e.*, a rapid, full, bounding pulse—surgical means with subsequent uterine venesection will do about all that is required. It is then, after the uterus is emptied, and then only, that its true value *may* become apparent. The statement made that when, under its influence, the pulse keeps slow, soft, and compressible, convulsions will not or do not occur, I wish to deny emphatically and in unmeasured terms, since I have seen terrible convulsive seizures when the pulse was 60 and alarmingly feeble. While writing this article, a case came under observation one-half hour after labor (induced) in which the pulse after a convulsion was 178, full and bounding. Hypodermics of full doses of veratrum sent the pulse down to 60 in one-half hour, and yet the worst fit the patient ever had occurred at this time. If, then, after labor, the pulse still remains full and bounding, it has been my custom to give the fl. extr. veratrum, from 5 to 10 min., and repeated in smaller doses every half-hour till the pulse is soft and slow. In order to overcome the depression which sometimes follows its administration, it has been my custom to add ¼ grn. morphine to the dose. Other measures fulfilling the same indications are belladonna, ammonia, atropine, and absolute rest. Veratrum should not be used in valvular lesions, or where the heart-muscle is weak, or in dilated or fatty heart. Under these conditions where, as a rule, the pulse is feeble and rapid, large doses of nitroglycerin are indicated.

Of venesection I speak as of veratrum. Where the condition is present to bleed, there is also a vital indication to empty the uterus; and if then venesection is indicated, let it be from that organ. All the measures before indicated are purely tentative. By their use there is ever present the hope so to improve our patient's condition that she may be tided over to such a time when labor may be induced or occur spontaneously.

It is a principle with me that when, after a short time, the urea estimation (above all clinical facts) shows no improvement, and certainly where the excretion decreases progressively, no matter what the other symptoms tell us, or what the condition of the patient may be, empty the uterus at once. A patient may feel good, suffer little, and yet the explosion may occur at any moment; and I defy anybody, when it does occur, to foretell the result.

But a few days ago I saw a primipara six and a half months pregnant, who passed 63 oz. of urine containing 5 per cent. of albumin and casts; specific gravity 1021, slowly falling to 1008, but amount of urine undiminished. Her pulse was soft; little œdema; and no constitutional symptoms; and yet because she excreted progressively less urea after two estimations down to 16 gme. in 24 hours, labor was terminated at once. Post-partum the most violent eclamptic attacks occurred, and death resulted in 12 hours.

After labor the treatment is to be continued as planned above. To control convulsions, chloroform is given to ward off attacks; chloral in large doses to allay seizures. Full doses of morphine, ½—1 grn., to quiet irritation, has been a useful measure in my hands. Ergot is not to be recommended after labor, from a theoretical standpoint, since it produces contraction of arterioles, causing an anemia of the brain·

Thus also from a physiological standpoint it is contraindicated.

IV. ACUTE TOXEMIA IN WHICH NEITHER ALBUMIN NOR CASTS APPEAR IN THE URINE; A TRUE URINEMIA.—The last subdivision is important from a diagnostic standpoint. To the careful observer it becomes evident that an intoxication is occurring, and upon his ability to make out the cause rests his success in treatment. When treated in time the course of the disease is mild; but where not recognized or when the cause is not found, or when found cannot be removed, we are face to face with as desperate a condition as falls to the lot of medical men. These are the malignant cases, and, do what you will, they nearly all die. To my mind the most frequent cause is pressure from the engaged head. In one case success was the result by placing the patient for a long period in a modified Trendelenburg posture (elevation of the end of a folding-bed), and were this to fail I would have been tempted to perform an external version, to change, if possible, the presentation from the head to the tranverse in order to overcome the presumed ureteral occlusion. But in any case it is safe and good treatment to empty the uterus at once, without very much prior treatment, depending upon active measures for success after the uterus is emptied.—*American Medico-Surgical Bulletin.*

